# Transcriptome profiling of *Capsicum annuum* using Illumina- and PacBio SMRT-based RNA-Seq for in-depth understanding of genes involved in trichome formation

**DOI:** 10.1038/s41598-021-89619-0

**Published:** 2021-05-13

**Authors:** Shenghua Gao, Ning Li, Juntawong Niran, Fei Wang, Yanxu Yin, Chuying Yu, Chunhai Jiao, Changxian Yang, Minghua Yao

**Affiliations:** 1 Hubei Key Laboratory of Vegetable Germplasm Enhancement and Genetic Improvement, Cash Crops Research Institute, Hubei Academy of Agricultural Sciences, Wuhan, 430070 Hubei China; 2 Key Laboratory of Horticultural Plant Biology (Ministry of Education), Huazhong Agricultural University, Wuhan, 430070 Hubei China; 3 Kasetsart University, Bangkok, 10900 Thailand

**Keywords:** Plant sciences, Gene expression analysis, Computational biology and bioinformatics

## Abstract

Trichomes, specialized epidermal cells located in aerial parts of plants, play indispensable roles in resisting abiotic and biotic stresses. However, the regulatory genes essential for multicellular trichrome development in *Capsicum annuum* L. (pepper) remain unclear. In this study, the transcript profiles of peppers GZZY-23 (hairy) and PI246331 (hairless) were investigated to gain insights into the genes responsible for the formation of multicellular trichomes. A total of 40,079 genes, including 4743 novel genes and 13,568 differentially expressed genes (DEGs), were obtained. Functional enrichment analysis revealed that the most noticeable pathways were transcription factor activity, sequence-specific DNA binding, and plant hormone signal transduction, which might be critical for multicellular trichome formation in hairy plants. We screened 11 DEGs related to trichome development; 151 DEGs involved in plant hormone signal transduction; 312 DEGs belonging to the MYB, bHLH, HD-Zip, and zinc finger transcription factor families; and 1629 DEGs predicted as plant resistance genes (PRGs). Most of these DEGs were highly expressed in GZZY-23 or trichomes. Several homologs of trichome regulators, such as *SlCycB2*, *SlCycB3,* and *H*, were considerably upregulated in GZZY-23, especially in the trichomes. The transcriptomic data generated in this study provide a basis for future characterization of trichome formation in pepper.

## Introduction

Plant trichomes, small specialized organs originating from epidermal cells, are one of the key factors serving as a buffer zone between the plant surface and the environment^1,2^. Trichomes are present in nearly all terrestrial plants and are classified into several different types, including non-glandular or glandular, unicellular or multicellular, and branched or unbranched^3^. The main role of trichomes is to provide both physical and chemical protection against biotic and abiotic stresses, such as herbivorous arthropods, pathogens, UV irradiation, and extreme temperature^4^. Glandular trichomes are considered biofactories that secrete or store many valuable metabolites, such as sclareol and artemisinin^5^. Furthermore, plant trichomes are an excellent model system for studying the molecular regulatory mechanisms of cell differentiation at the single-cell level, owing to their simple structure^6^.

The molecular mechanism of unicellular trichome formation has been well characterized, especially in the leaf trichomes of the model plant *Arabidopsis thaliana*^6,7^. In *Arabidopsis*, trichome differentiation is thought to be regulated by an activator-inhibitor mechanism. The crucial positive transcription factors (TFs) include GLABRA1 (GL1), its functionally equivalent counterparts WEREWOLF (WER) and MYB23, which act as R2R3 type-MYB transcription factors^8,9^; GLABRA3 (GL3), ENHANCER OF GLABRA3 (EGL3), TRANSPARENT TESTA (TT8), and MYC-1, which act as basic helix-loop-helix (bHLH) proteins^10–12^; and TRANSPARENT TESTA GLABRA1 (TTG1), which acts as a WD40-repeat protein^13^. Moreover, the negative transcription factors include TRIPTYCHON (TRY), CAPRICE (CPC), ENHANCER OF TRYAND CPC1 (ETC1), ECT2, ETC3, CAPRICE-LIKE MYB3, and TRICHOMELESS1 and TRICHOMELESS2 (TCL1 and TCL2, respectively), which belong to the small, single-repeat MYB family^14–18^. The positive transcription factors combine to form an MYB-bHLH-WD40 (MBW) trimeric complex that stimulates epidermal cells to differentiate into trichomes by activating the expression of the downstream activators homeodomain-leucine zipper (HD-Zip) transcription factor, *GLABRA2* (*GL2*)^14^. In the neighboring cells of trichome, the negative transcription factors compete with R2R3 type-MYB for binding to the bHLH-WD40 complex, forming an inactivating trimeric complex, which cannot promote *GL2* expression, to suppress trichome initiation^19,20^.

Compared with studies on unicellular trichomes, studies on the development and regulatory networks of multicellular trichomes in plants are still limited. Only a few genes controlling multicellular trichome formation are characterized. The MYB transcription factor *MIXTA* in snapdragon (*Anthirrhinum majus* L.), as well as two other homologs, *AmMYBML1* and *CotMYBA*, which are from snapdragon and cotton, respectively, can activate multicellular trichome formation when ectopically expressed in tobacco^21,22^. The overexpression or repression of *AaMIXTA1* can result in an increase or decrease in the number of glandular secretory trichomes in *Artemisia annua*, respectively^23^. An AP2 transcription factor, TRICHOME AND ARTEMISININ REGULATOR 1, positively regulates the development of multicellular trichome in *A. annua*^24^. The *Woolly* (*Wo*) gene, which encodes a HD-Zip IV transcription factor, is responsible for multicellular trichome formation in tomato; it also induces multicellular trichome formation when ectopically expressed in tobacco^25,26^. Furthermore, HD-ZIP IV transcription factor-encoding genes, *OCL4* in Z*ea mays*, *Tril* in *Cucumis sativus*, *GLABROUS* (*CmGL*) in *Cucumis melo* L, *AaHD8* in *Artemisia annua* have been confirmed to be involved in multicellular trichome formation^27–30^. HD-ZIP I transcription factor-encoding gene *CsGL1*, positively regulates the formation of trichomes and fruit spines in cucumber^31^. The C2H2 zinc-finger protein-encoding genes are also involved in trichome formation, such as *H* in tomato and its homolog *CaH* in pepper^32^. The B type cyclin gene, *SlCycB2*, as well as its homologs, *SlCycB3* in tomato, *NtCycB2* in tobacco, and *AtCycB2* in *Arabidopsis thaliana*, promotes trichome formation when expressed in tomato and in tobacco^33^. However, the overexpression of neither *AtCycB2* nor *Wo* in *Arabidopsis thaliana* has an effect on trichome formation, indicating that the initiation of multicellular and unicellular trichome formation may be regulated by different pathways^25^. So far, the developmental process of multicellular trichomes and the underlying regulatory molecular mechanisms remain unclear.

Pepper (*Capsicum annuum* L.), one of the most commercially important vegetable crops worldwide, is used for both medicinal and culinary purposes^34^. The trichomes on pepper leaves, or stems, are highly specialized structures originating from the epidermal cells, and have recently attracted research attention for their effect on biotic and abiotic stress responses in plants. Similar to those in tomato, the trichomes in pepper are multicellular. Previous studies have shown that the pepper trichome locus 1 (*Ptl1*) is located in the same region as the *H* (*hair*) locus in tomato^32,35^. These results indicate genetic loci controlling trichome formation is conserved in Solanaceae. Thus, research on pepper trichomes is needed to analyze the mechanism of multicellular trichome formation.

Illumina RNA sequencing (RNA-Seq) has become an extremely powerful tool for revealing the relationships between genotypes and phenotypes, thereby increasing our understanding of the underlying pathways and genetic mechanisms controlling many processes, including cell growth, development, and immune regulation^26,36^. In plants, RNA-Seq has been used to study global expression profiles and reveal signal transduction pathways involved in trichome formation in plants such as tobacco and cucumber^26,37^. Furthermore, a few studies on transcription profiles and gene function analysis in pepper have been conducted. For example, the gene expression profiles of brassinosteroids (BRs), which induce chilling tolerance, have been analyzed in pepper by RNA-Seq, indicating that BR responses to the chilling stress in pepper involved the activation of extensive transcriptional activities, signaling transduction, and modulation of metabolic homeostasis^38^. Candidate enzyme-encoding genes involved in capsaicin biosynthesis are identified by a combination of RNA-Seq and digital gene expression profiling (DGE) analyses at different fruit developmental stages in the pepper Guijiangwang^39^. The fundamental limitation for Illumina RNA-Seq is the short sequencing product, which requires assembly and leads to a small proportion of assembled transcripts, and to misassembly as well. In recent years, the full-length transcriptome has been used as an effective approach to obtain high-quality transcript sequences. Single molecule real-time (SMRT) sequencing developed by Pacific Biosciences (PacBio) can help achieve full-length sequencing without full-length post-sequencing assembly^40,41^. To our knowledge, the high error rate of PacBio reads seriously offsets the advantages of long reads, whereas, Illumina RNA-Seq reads can effectively eliminate the high error rate of SMRT sequencing^42^. Hence, the combination of SMRT sequencing and Illumina RNA-Seq is a preferable method, and has been used for whole-transcriptome profiling in many plants^43–45^. However, to the best of our knowledge, there are no reports about the gene expression profiles for trichome formation in pepper.

In this study, we investigated the trichomes on GZZY-23 (hairy) and PI246331 (hairless) pepper plants were characterized. We further performed comparative transcriptome profiling analysis to identify genes associated with multicellular trichome formation using Illumina HiSeq 2500 sequencing and SMRT RNA-Seq technology. A series of candidate genes containing key transcription factors were identified, which might be involved in the development of multicellular trichomes. The study provides comprehensive transcriptomic information regarding trichome development in pepper leaves and further promotes our understanding of the detailed molecular mechanisms of multicellular trichome differentiation and the subsequent cell proliferation.

## Results

### Trichome phenotypic analyses

**Figure 1:**
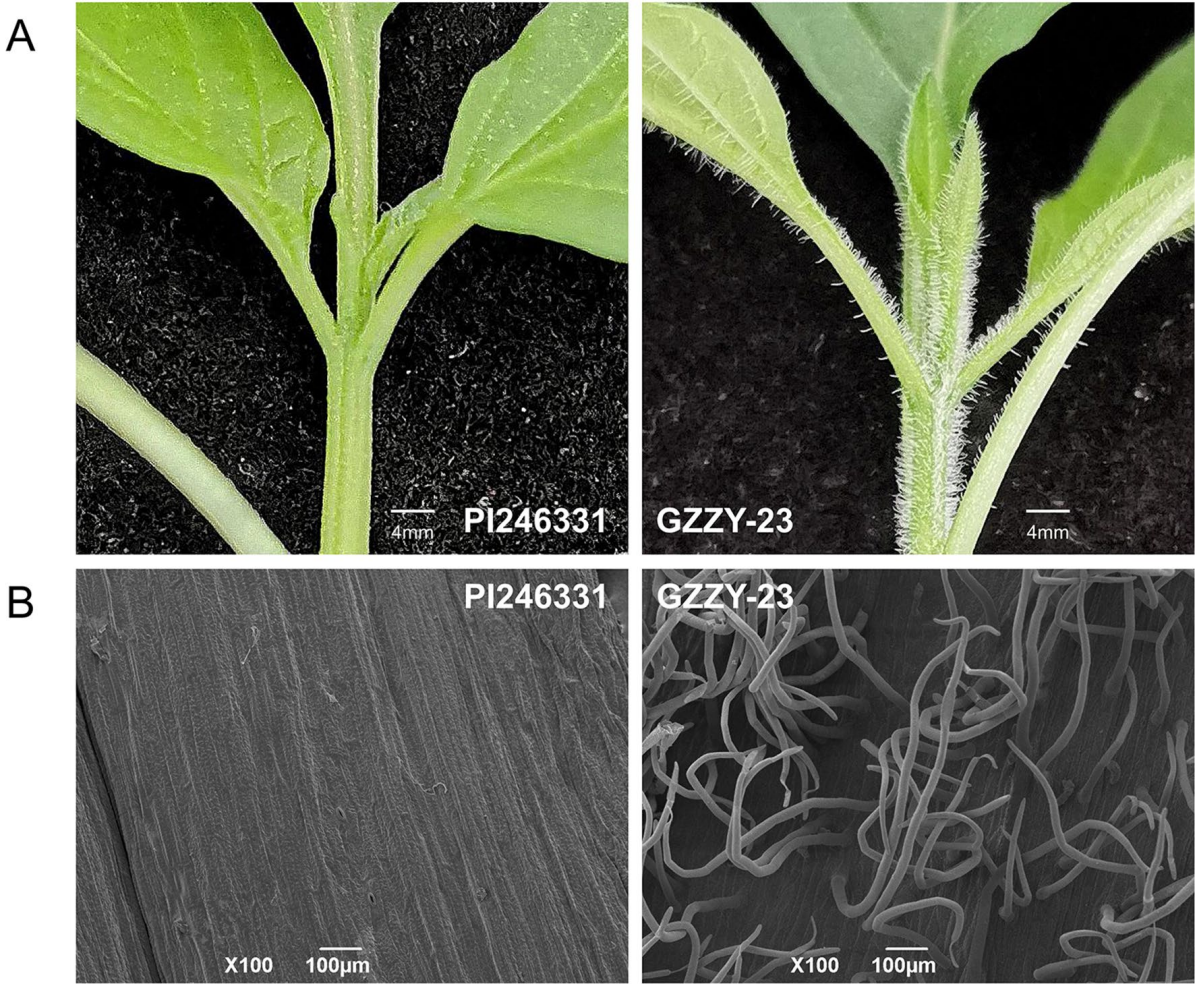
Phenotypes of trichomes in pepper varieties PI246331 and GZZY-23. **(A)** Digital photograph of growth tips and stems of PI246331 and GZZY-23 at four-leaf stage. Scale bar, 4 mm. **(B)** Scanning electron micrographs of PI246331 and GZZY-23 stems at four-leaf stage. Scale bar, 100 μm.

Trichomes are specialized epidermal cells, which are present on the leaves, stems, carpopodium, and calyx of pepper^3^. Here, a new variety of pepper, designated as GZZY-23, was identified, which exhibits large, dense, and long trichomes on the surface of the stems and leaves (Fig. 1A). However, another pepper variety PI246331 was glabrous on the leaves and stems (Fig. 1A).

To characterize the trichome phenotypes, we conducted a scanning electron microscopy (SEM) assay. We found that long macro-trichomes and sporadic short trichomes cover the stem surface of GZZY-23 (Fig. 1B). PI246331 had sporadic short trichomes on the stems and presented glabrous phenotype (Fig. 1B). Different from the trichomes in *Arabidopsis* and tomato^46,47^, the long trichomes on GZZY-23 stems were simple multicellular trichomes, which had no branches, glands, or prickles (Fig. 1A,B).

### Sequencing and de novo transcriptome assembly

To identify differentially expressed genes (DEGs) that possibly led to multicellular trichome formation, nine cDNA libraries of PI246331, GZZY-23, and Trichome were sequenced by Illumina RNA-seq, and one cDNA library was constructed by SMRT technology. For SMRT sequencing, the Pacific Biosciences Sequel platform was used to obtain 27,748,483 subreads. 299,864 circular consensus sequence (CCS) reads were identified, with an average length of 1654 bp and an N50 length of 1988 bp, including 226,497 full length (FL) reads, 192,252 full-length non-chimeric (FLNC) reads, and 192,094 FLNC reads with polyA. The average length of the FLNC reads with polyA was 1301 bp, and its N50 length was 1547 bp (Table S1).

For Illumina sequencing, 54.37 Gb clean reads were obtained with an average of 6.04 Gb for each sample, after removing low quality reads and trimming adapter sequences (Table S2). The average Q30 contents were 88.1% to 91.6%, and GC contents were 41.9% to 44.2%, respectively (Table S2). These data indicate that the sequencing results were accurate. To assess the quality of the RNA-Seq data, the reads were mapped to the *Capsicum annuum*. L Zunla-1 reference genome. Among them, 87%-93% clean reads in each sample were successfully mapped to the reference genome (Table S2). Of these, the proportion of mapped reads among PI246331, GZZY-23, and Trichome samples was 71% to 84% of the unique mapping ratio. The percentage of the unique mapping reads was above 75% in each sample, suggesting that the RNA-Seq libraries exhibited high quality. In addition, correlation efficient of gene expression among three biological replicates indicated high repeatability (Table S3). These results demonstrated that the assembly quality of RNA-Seq data was satisfactory.

### Gene structure and function annotation

In the present study, 192,094 FLNC reads with polyA were used for identifying gene loci and transcripts based on the reference genome of Zunla-1. The removal of redundant transcripts reduced the number of transcripts to 29,854, corresponding to 18,785 gene loci, and each represented a unique full-length transcript (Table 1). Furthermore, 19,611 novel transcripts were identified, of which 15,990 (81.54%) transcripts were predicted as open reading frames (ORFs) (Table S4). By comparing with 35,336 genes annotated in the pepper genome, 4743 novel genes with 5713 isoforms were identified based on SMRT sequencing in the present study (Tables 1 and S5). The number of transcripts greater than 2 kb generated by SMRT sequencing was significantly higher than that produced from the reference genomes (Table 1). Furthermore, 3705 of the 5713 novel isoforms were annotated according to the National Center for Biotechnology Information (NCBI) nonredundant (NR), Swiss-Prot, gene ontology (GO), clusters of euKaryotic orthologous genes (KOG) protein databases, and the Kyoto Encyclopedia of Genes and Genomes (KEGG) pathway database (Table S4). A total of 1427 (24.98%), 1057 (18.50%), 595 (10.41%), 3698 (64.73%), and 1877 (32.85%) transcripts were assigned to the GO, KO, KOG, NR, and Swiss-Prot databases, respectively (Table S5).

**Table 1:** Statistics of gene loci and the isoforms annotated from SMRT sequencing data.

Feature	Annotation.loci.len	PacBio.loci.len
Loci	35,336	18,785
Loci < 1 K	21,954(62.13%)	4437(23.62%)
Loci 1–2 K	10,055(28.46%)	7544(40.16%)
Loci 2–3 K	2371(6.71%)	4378(23.31%)
Loci ≥ 3 K	956(2.71%)	2426(12.91%)
Total isoform	35,336	29,854

*Loci* gene loci, *Loci < 1 K, Loci 1–2 K, Loci 2–3 K and Loci ≥ 3 K* the length of gene sequence, *Total isoform* total transcripts, *Annotation.loci.len* the number of gene loci annotated on the genome, *PacBio.loci.len* the number of gene loci annotated based on PacBio data.

Alternative splicing (AS) can enhance transcriptome plasticity and proteome diversity. In plants, AS occurs at different stages of development^48^. It is well known that long reads generated by SMRT sequencing platform are suitable for widely and accurately identifying AS forms^49,50^. In this study, 4427 AS events were identified in the transcripts by ASTALAVISTAA Stalavista^51^. The main types of AS included exon skipping (ES), intron retention (IR), alternative donor sites (AD), and alternative acceptor sites (AA) (Table S6). Among the main types of alternatively spliced transcripts, IR was found to be predominant, accounting for 26.84% of the AS transcripts, followed by AA (18.86%), ES (12.26%), and AD (8.53%) (Table S6).

Long non-coding RNAs (LncRNAs), which are not translated into proteins, widely exist in plants and play important roles in plant growth and development^52^. Here, 2227 LncRNAs were predicted (Table S7). Additionally, 10,327 alternative polyadenylation (APA) sites in 5947 genes were identified using full-length transcriptome APA detection software Tapis^53^ (Table S8).

### Gene expression analysis based on Illumina and SMRT data

**Figure 2:**
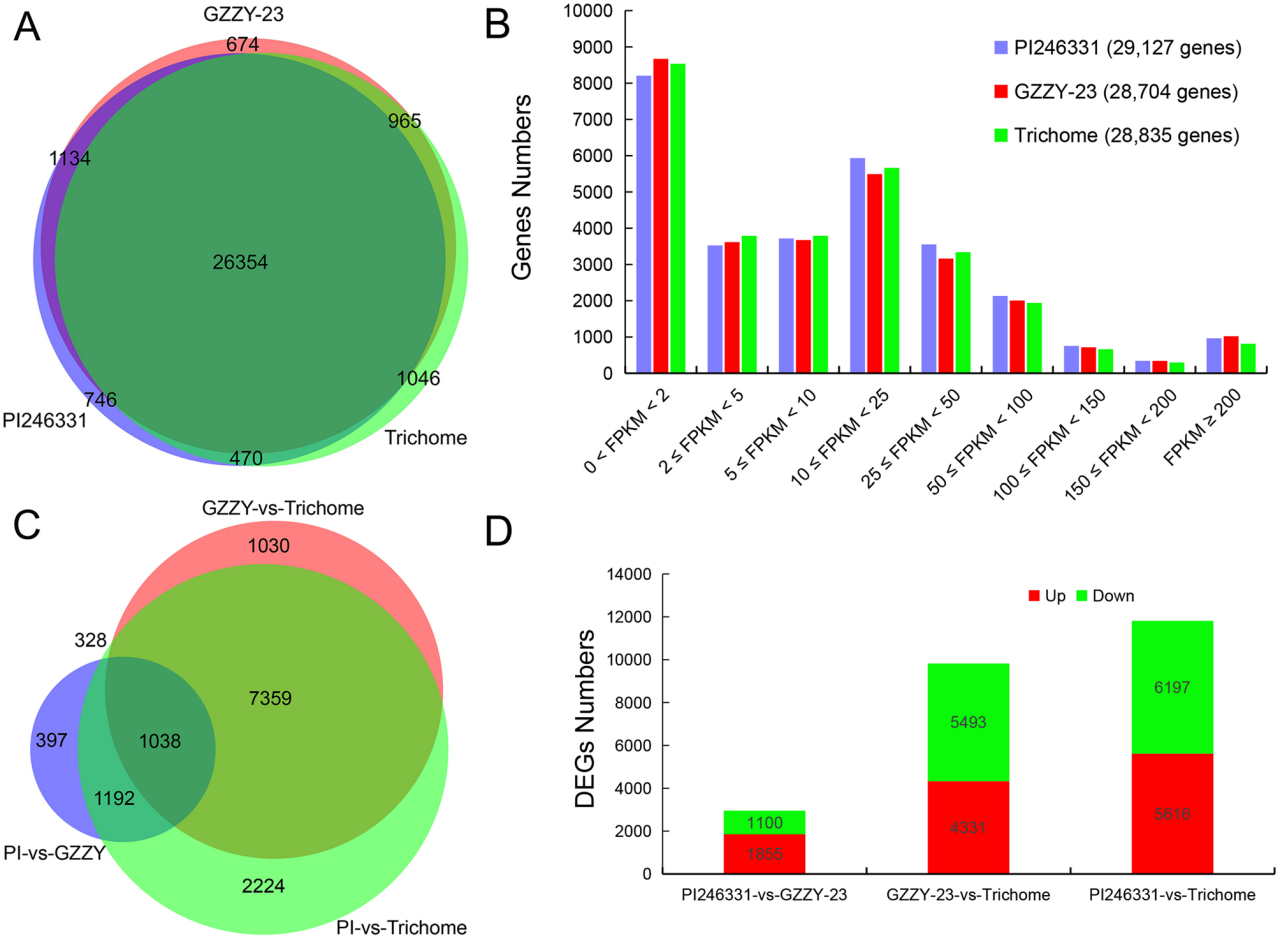
Statistics and Venn diagram analysis of the expressed genes and DEGs in different cDNA libraries. **(A)** Venn diagram of the expressed genes for each library. **(B)** The frequency represents the number of genes per category according to the FPKM expression value. The number of total considered expressed genes for each moment is presented in brackets. **(C)** Venn diagram illustrating DEGs in the different comparisons. PI, PI246331; GZZY, GZZY-23. **(D)** Number of DEGs examined in different comparisons.

194,591,984 clean read pairs produced by Illumina sequencing were aligned to the newly constructed transcript library using Bowtie software^54^. The gene expression patterns of PI246331, GZZY-23, and Trichome samples were determined using the fragments per kilobase million (FPKM) values and RSEM software^55^. The results revealed 28,704, 29,127, and 28,835 genes expressed in PI246331, GZZY-23, and Trichome, respectively (Fig. 2A). Among these expressed gene clusters, the FPKM values of 75% of the genes were < 25, approximately (Fig. 2B), and those of approximately 3% of the genes were > 200 (Fig. 2B). It was clear that the gene expression levels in different materials displayed a similar trend (Fig. 2B). The gene expression distribution analysis showed that among the 31,389 expressed genes, 26,354 (84%) genes were expressed in all three materials, whereas a small number of genes were expressed only in one condition (2.4% of PI246331, 2.1% of GZZY-23, and 3.3% of Trichome) (Fig. 2A). Furthermore, 965 (3.1%) genes were expressed in both GZZY-23 and Trichome, but not expressed in PI246331 (Fig. 2A).

DEGs were statistically evaluated according to the DESeq2 method^56^. By comparing in pairs, 13,568 DEGs were obtained, of which 2955 DEGs were discovered in PI246331 vs. GZZY-23, 9824 DEGs in GZZY-23 vs. Trichome, and 11,813 DEGs in PI246331 vs. Trichome (Fig. 2C,D). Among these DEGs, the expression of 2049 (1176 upregulated and 873 downregulated) DEGs was both upregulated or downregulated in GZZY-23 and Trichome compared with those in PI246331, respectively. Furthermore, 3722 and 4608 DEGs were both upregulated or downregulated in Trichome compared with those in GZZY-23 and PI246331, respectively (Tables S9, S10, and S11). Moreover, 1038 DEGs overlapped in the three comparisons (Fig. 2C), including 521 and 474 DEGs most highly expressed in GZZY-23 and Trichome, respectively (Tables S9, S10, and S11). It was obvious that there were more genes upregulated in the three comparisons, indicating that many genes involved in trichome formation were highly expressed in GZZY-23, especially in Trichome.

### GO and KEGG pathway analyses of DEGs

**Figure 3:**
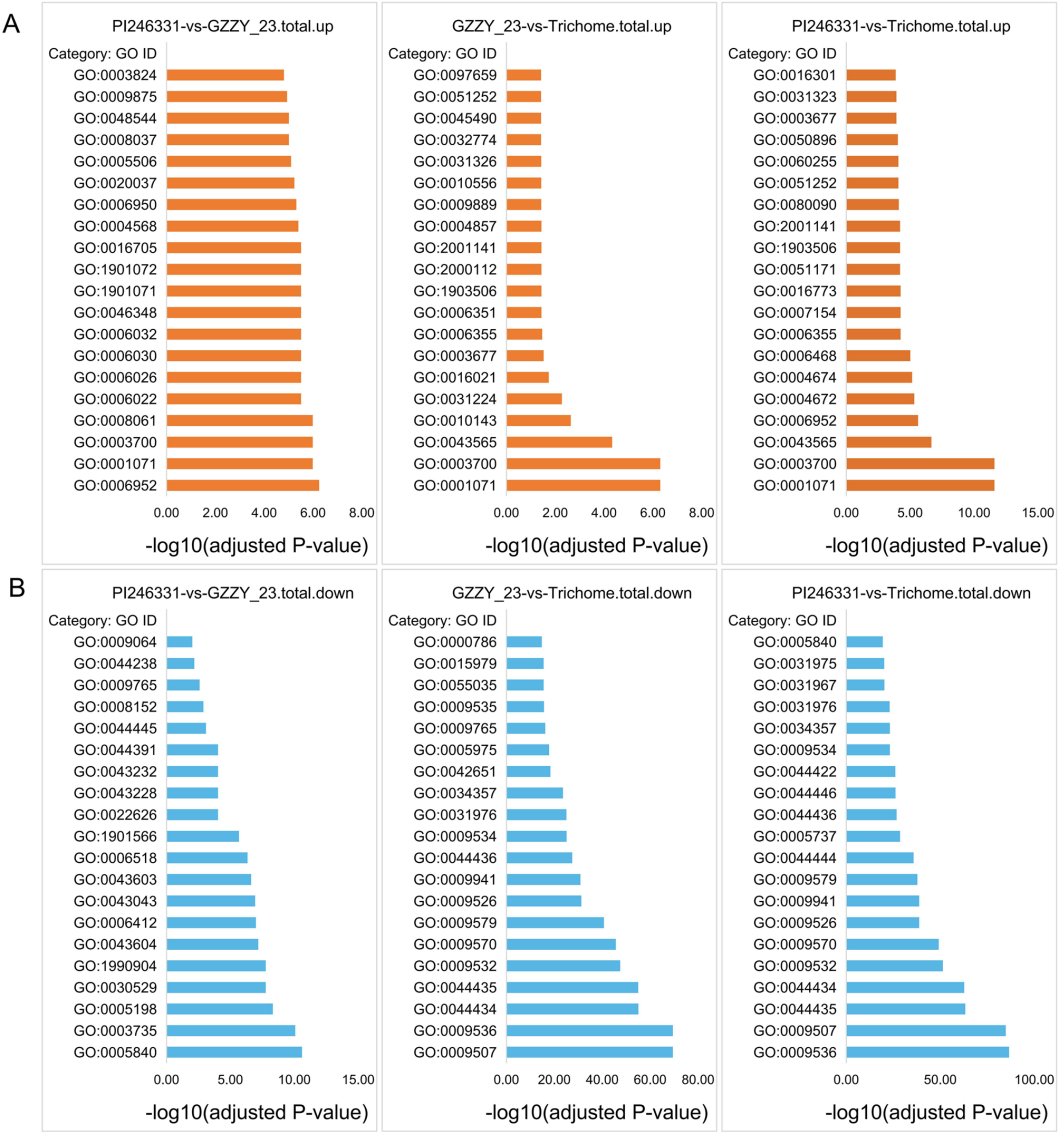
Enrichment analysis of deferentially expressed genes. **(A)** The top 20 significantly enriched GO terms for the upregulated DEGs in different comparisons. **(B)** The top 20 significantly enriched GO terms for the downregulated DEGs in different comparisons. The abscissa is the logarithm of the significant enrichment P value with base 10, and the ordinate is the GO ID.

To explore the biological functions of DEGs in PI246331, GZZY-23, and Trichome, Gene Ontology (GO) enrichment analysis was carried out. In total, 8285 of 13,568 DEGs in the three comparisons (PI246331 vs. GZZY-23, GZZY-23 vs. Trichome, and PI246331 vs. Trichome groups) were annotated with GO terms and assigned to three categories: biological process (BP), cellular component (CC), and molecular function (MF) (Table S12). The upregulated DEGs in the three comparisons were commonly enriched in transcription factor activity, sequence-specific DNA binding (GO: 0003700), sequence-specific DNA binding (GO: 0043565), and nucleic acid binding transcription factor activity (GO: 0001071) in the MF category (Fig. 3A; Table S12). The downregulated DEGs were commonly enriched in the peptide biosynthetic process (GO: 0043043) and photosynthesis light harvesting (GO: 0009765) (Fig. 3B; Table S12). Furthermore, the upregulated DEGs in the comparison PI246331 vs. GZZY-23 were specifically enriched in metabolic process (GO:0008152), response to stress (GO:0006950), defense response (GO:0006952), monocarboxylic acid metabolic process (GO:0032787), and secondary metabolite biosynthetic process (GO:0044550) in the BP category, and in catalytic activity (GO:0003824), oxidoreductase activity (GO:0016491), and tetrapyrrole binding (GO:0046906) in the MF category (Fig. 3A). The DEGs downregulated in Trichomes compared with those in PI246331 and GZZY-23 were enriched in the carbohydrate catabolic process (GO: 0016052), carbohydrate metabolic process (GO: 0005975), photosynthesis (GO: 0015979), light harvesting in photosystem I (GO: 0009768), and plastid thylakoid (GO: 0031976) (Fig. 3B).

**Figure 4:**
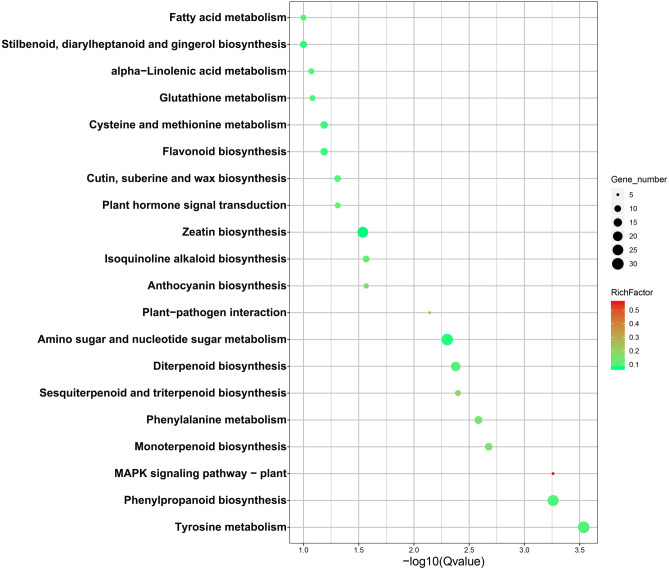
Top 20 KEGG enrichment pathways by FDR in PI246331 vs. GZZY-23. Rich factor is the proportion of the deferentially expressed genes in the pathway. The higher the rich factor, the higher the degree of enrichment. The FDR is the false discovery rate, ranging from 0 to 1; the closer the value to zero, the more significant enrichment.

In addition, the KEGG pathway analysis was performed to determine the functional networks of biological interactions. In this study, 10,574 DEGs in PI246331 vs. GZZY-23, GZZY-23 vs. Trichome, and PI246331 vs. Trichome were assigned to 128 KEGG pathways (Table S13). The top 20 KEGG pathways enriched by upregulated or downregulated DEGs are shown in Fig. 4 and Table S13. The upregulated DEGs were largely enriched in phenylpropanoid biosynthesis (ko00940), MAPK signaling pathway-plant (ko04016), monoterpenoid biosynthesis (ko00902), phenylalanine metabolism (ko00360), sesquiterpenoid and triterpenoid biosynthesis (ko00909), diterpenoid biosynthesis (ko00904), amino sugar and nucleotide sugar metabolism (ko00520), and plant-pathogen interaction (ko04626) in PI246331 vs. GZZY-23 (Fig. 4). Similarly, many of these pathways were enriched in GZZY-23 vs. Trichome and PI246331 vs. Trichome, like plant-pathogen interaction (ko04626), MAPK signaling pathway-plant (ko04016), and plant hormone signal transduction (ko04075). However, the downregulated DEGs were mainly enriched in photosynthesis-antenna proteins (ko00196) in the three comparisons (Table S13). Carbon fixation in photosynthetic organisms (ko00710), starch and sucrose metabolism (ko00500), glyoxylate and dicarboxylate metabolism (ko00630), carbon metabolism (ko01200), biosynthesis of amino acids (ko01230), cysteine and methionine metabolism (ko00270), the pentose phosphate pathway (ko00030), and aminoacyl-tRNA biosynthesis (ko00970) were also enriched by the downregulated DEGs in GZZY-23 vs. Trichome and PI246331 vs. Trichome (Table S13).

### DEGs and TFs related to trichome development and validation by qRT-PCR

**Figure 5:**
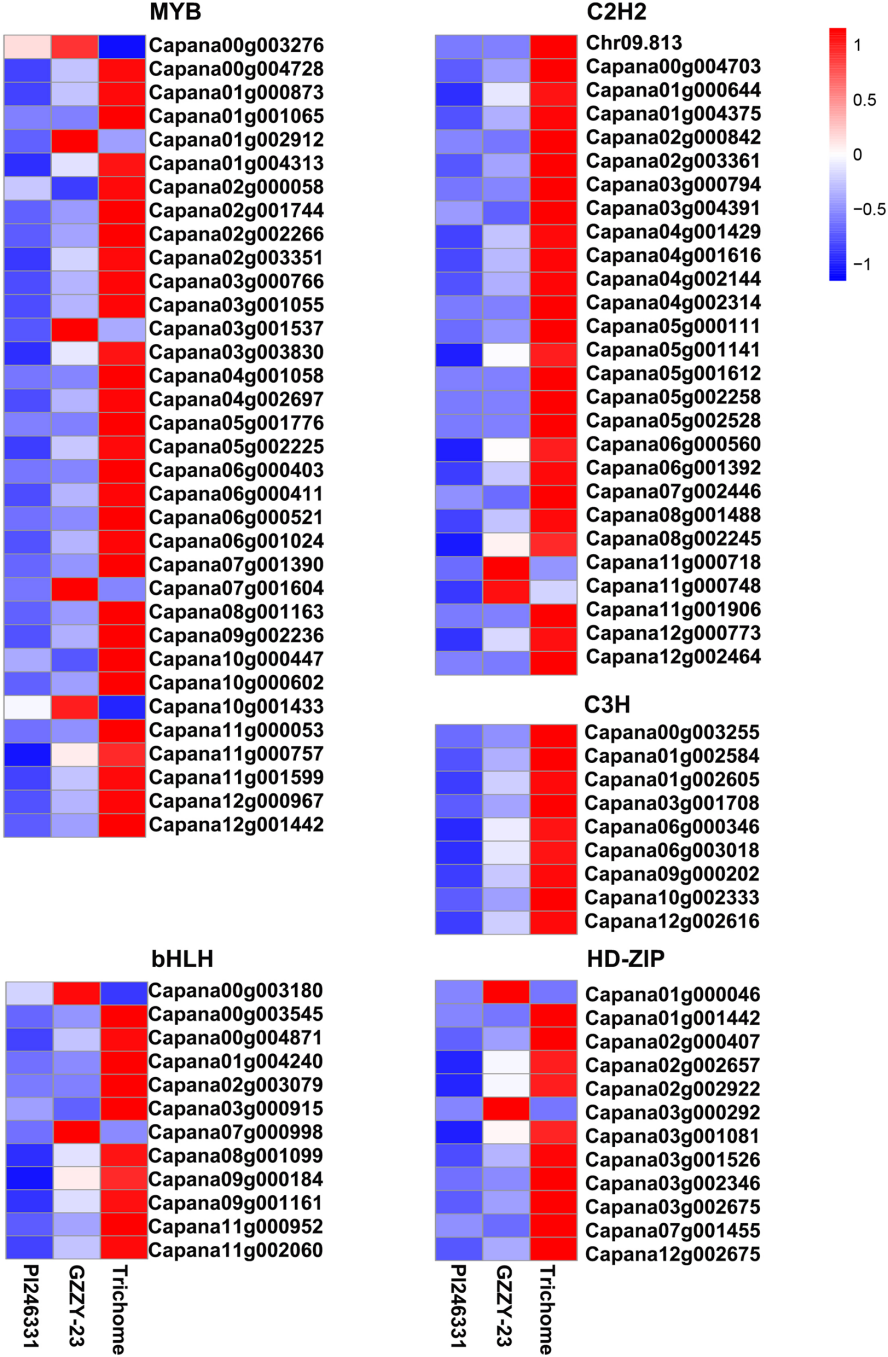
Expression profiles of the upregulated transcription factors belonging to the MYB, bHLH, HD-ZIP, and zinc finger proteins in GZZY-23 and Trichome. The redder the bars, the higher the gene expression level.

**Figure 6:**
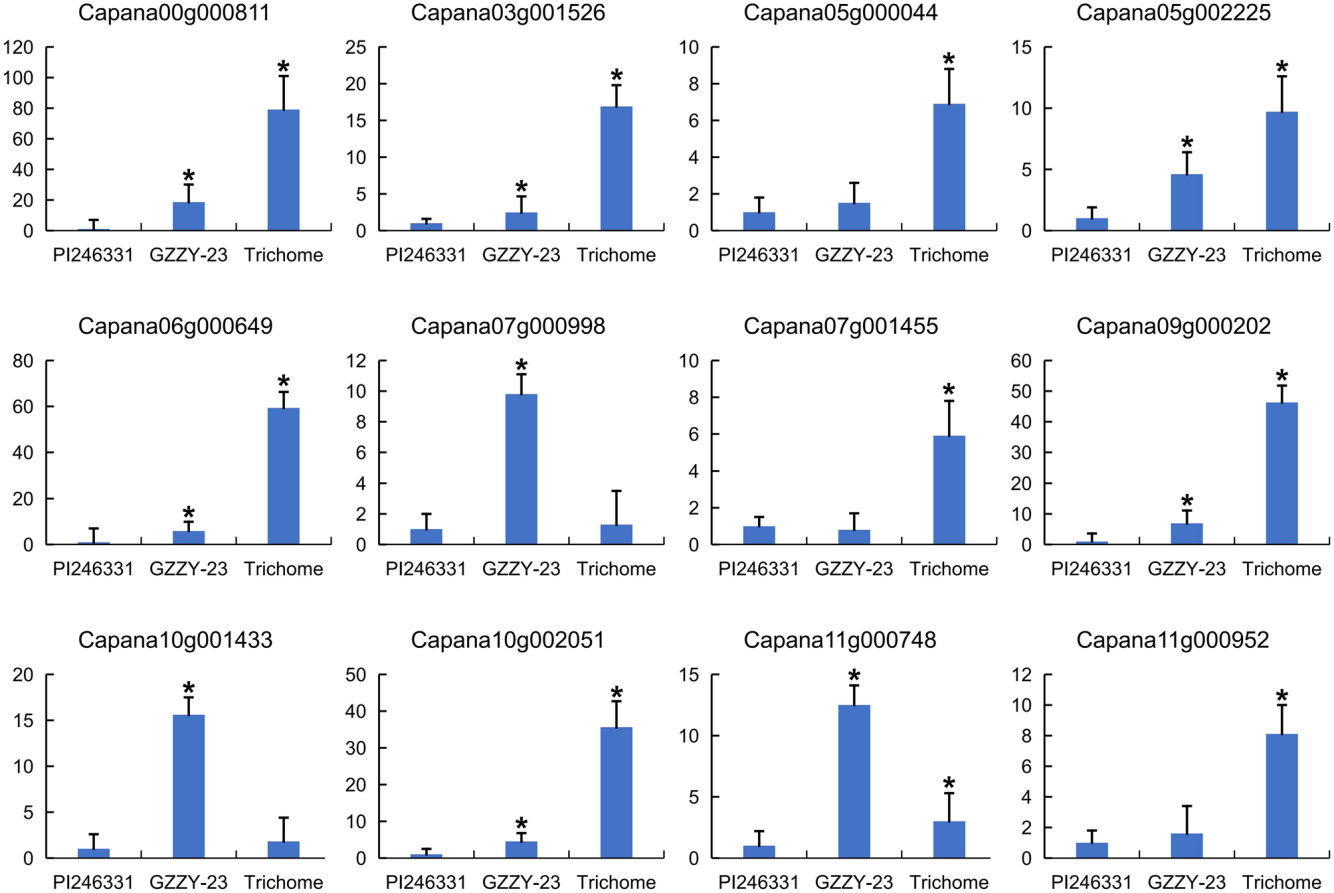
QRT-PCR analysis of the differentially expressed genes among PI246331 growth tips, GZZY-23 growth tips, and trichomes**.** The abscissa is pepper tissues, and the ordinate is gene relative expression level. The relative expression level of genes in growth tips of PI246331 is used as a control. Data are presented as means ± SEM. Asterisks indicate statistically significant differences (* P < 0.05 by Dunnett test, n = 3).

To explore the possible genes controlling trichome development in pepper, many functional genes were identified. We identified 11 DEGs that may be involved in trichome differentiation (GO: 0010026) and trichome morphogenesis (GO: 0010090) in the comparisons of PI246331 vs. GZZY-23, GZZY-23 vs. Trichome, and PI246331 vs. Trichome (Table S14). Among these, all genes were identified in comparisons GZZY-23 vs. Trichome and PI246331 vs. Trichome, except for *Chr06.1236* and *Capana03g000947*. Interestingly, *Capana07g000392* was common DEG in the three comparison groups. However, *Capana03g000947* was identified in comparisons PI246331 vs. GZZY-23 and PI246331 vs. Trichome. Chr06.1236 was identified in the comparison PI246331 vs. GZZY-23 (Tables S9, S10, and S11). Moreover, 312 DEGs encoding MYB, bHLH, HD-Zip, and zinc finger transcription factors were also discovered. Among them, 72, 249, and 265 DEGs were identified in the comparisons PI246331 vs. GZZY-23, GZZY-23 vs. Trichome, and PI246331 vs. Trichome, respectively (Table S15). In addition, more than half of the DEGs in PI246331 vs. GZZY-23 were upregulated (Table S15). However, most DEGs in GZZY-23 vs. Trichome and PI246331 vs. Trichome were downregulated (Table S15). Interestingly, 11 and 83 DEGs were highly expressed in GZZY-23 and trichomes, respectively (Fig. 5; Table S15). Moreover, in the present study, *CaH* (*Capana00g000811*), *CaCycB2* (*Capana10g002051*, *SlCycB2* homolog), and *CaCycB3* (*Capana06g000649*, *SlCycB3* homolog) were also upregulated in GZZY-23, especially in Trichome (Fig. 6; Table S15).

To verify the sequencing data, 12 DEGs, including *CaH* (*Capana00g000811*), *CaCycB2* (*Capana10g002051*), and *CaCycB3* (*Capana06g000649*), were selected for qRT-PCR verification. As expected, *Capana07g000998*, *Capana10g001433*, and *Capana11g000748* were highly expressed in GZZY-23 (Fig. 6). The expression of the other DEGs was the highest in the trichomes. Generally, the expression patterns determined by qRT-PCR were consistent with those obtained by RNA-Seq (Fig. 6), which confirmed the accuracy of the RNA-Seq results reported in this study.

### DEGs related to signal transduction

**Figure 7:**
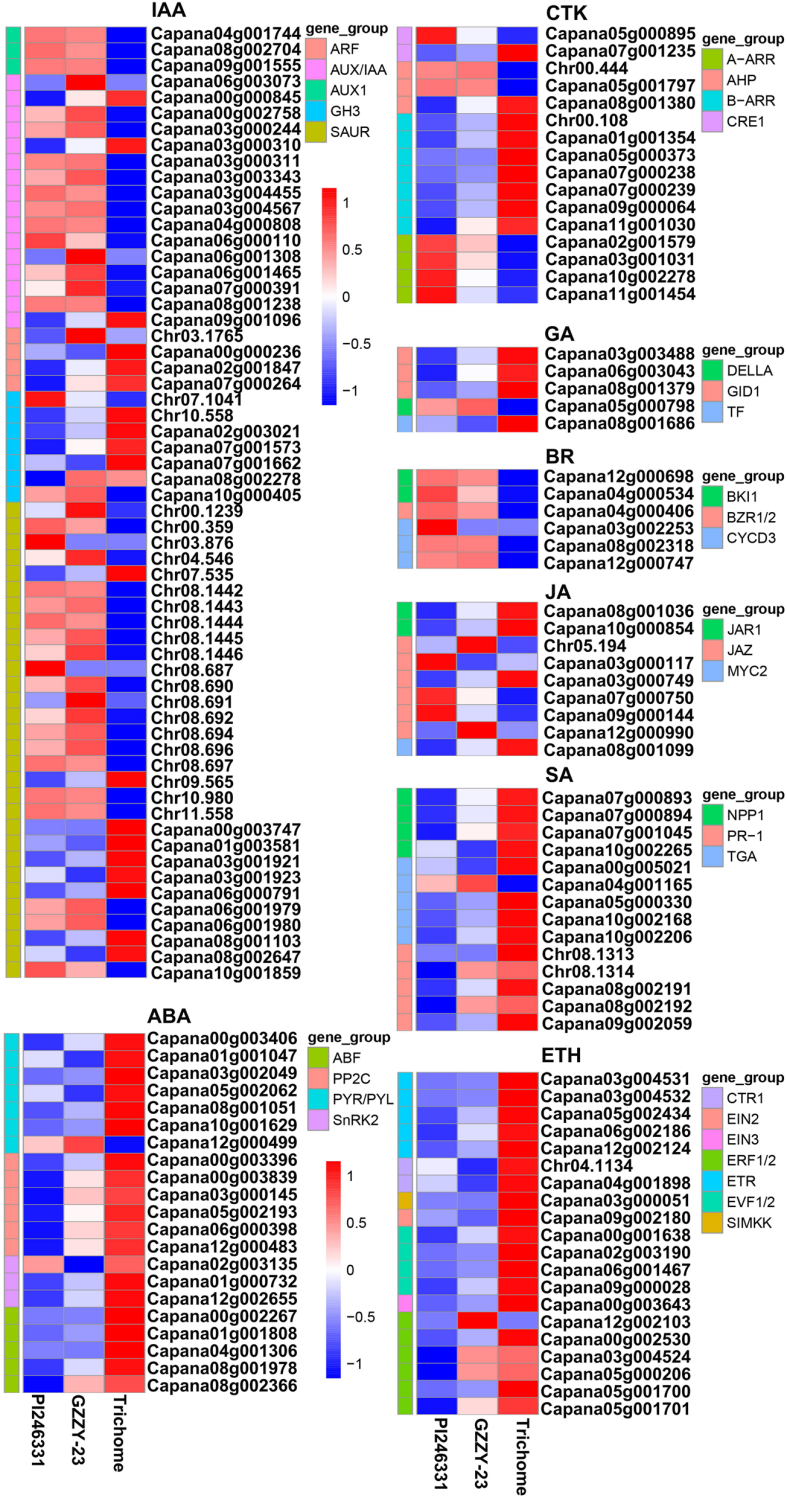
Expression profiles of DEGs related to plant hormone signal transduction in PI246331 growth tips, GZZY-23 growth tips, and Trichome. The redder the bars, the higher the gene expression level.

In this study, a total of 151 DEGs were enriched in plant hormone signal transduction pathway in the three comparisons (Fig. 7). In auxin (indoleacetic acid, IAA) signal transduction, most DEGs encoding auxin response factor (ARF) and auxin responsive GH3 gene family (GH3) were upregulated in Trichome, whereas DEGs encoding auxin responsive protein IAA (Aux/IAA) and auxin influx carrier (AUX1) were downregulated in Trichome (Fig. 7). In cytokinine (CTK) signal transduction, DEGs encoding two-component response regulator ARR-A family (ARR-A) were upregulated in PI246331, whereas they were downregulated in Trichome. DEGs encoding two-component response regulator ARR-B family (ARR-B) were upregulated in Trichome (Fig. 7). In gibberellin (GA) signal transduction, DEGs encoding GA receptor GID1 and phytochrome-interacting factor 4 (TF) were upregulated in Trichome (Fig. 7). In brassinosteroid (BR) signal transduction, all DEGs were downregulated in Trichome (Fig. 7). In jasmonic acid (JA) signal transduction, 1 and 5 DEGs encoding jasmonate ZIM domain-containing protein (JAZ) were upregulated and downregulated in Trichome, whereas DEGs encoding jasmonic acid-amino synthetase (JAR1) and jasmonate ZIM domain-containing protein (JAZ) were upregulated (Fig. 7). Moreover, all DEGs involved in abscisic acid (ABA), ethylene (ETH), and salicylic acid (SA) signal transduction, were upregulated in trichomes, except two DEGs encoding the ABA receptor PYR/PYL family (PYR/PYL) and transcription factor TGA (Fig. 7).

### DEGs related to plant resistance

Plant trichomes play an essential role in the protection of plants against biotic and abiotic stresses. In this study, we identified 1782 plant resistance genes (PRGs) in PI246331, GZZY-23, and Trichome, which were divided into 23 categories, including RLP, TNL, RLK, CNL, and RLK-GNK2 (Tables 2 and S17). Among these, 1629 PRGs were obviously differentially expressed in PI246331, GZZY-23, and Trichome. A total of 314, 991, and 1152 DEGs were identified in PI246331 vs. GZZY-23, GZZY-23 vs. Trichome, and PI246331 vs. Trichome, respectively, which were divided into 22 categories. Differentially expressed PRGs were mainly classified into RLK, NL, RLP, N, CNL, T, and TNL-OT categories (Table 2). Furthermore, differentially expressed PRGs were enriched in TNL categories in the comparisons GZZY-23 vs. Trichome and PI246331 vs. Trichome (Table 2). Additionally, most of these differentially expressed PRGs were upregulated in each comparison (Table S16).

**Table 2:** Distribution of differentially expressed plant resistance genes (PRGs) in PI246331, GZZY-23, and trichome.

Category	PI246331 vs. GZZY-23		GZZY-23 vs. trichome		PI246331 vs. trichome	
	Up	Down	Up	Down	Up	Down
CN	1	2	12	4	13	6
CNL	18	8	65	35	78	43
CNL-R1	1	1	5	1	8	1
CNL-RPW8	0	0	2	0	2	0
L	0	2	7	0	6	1
Mlo-like	4	0	6	3	9	3
N	23	10	79	47	96	48
NL	44	4	69	60	92	49
NL-R1	0	0	1	0	2	0
Other	10	0	8	9	14	8
Pto-like	6	1	11	4	14	2
RLK	54	7	62	62	86	56
RLK-GNK2	7	1	19	13	22	13
RLK-Malectin	0	0	0	5	0	5
RLK-Pto-like	0	0	0	2	0	1
RLP	28	18	88	126	112	129
RLP-Malectin	0	0	0	1	0	1
T	27	2	39	24	62	22
TN	0	1	0	0	0	0
TNL	0	0	65	53	83	60
TNL-OT	25	8	4	0	4	0
TNL-TNL	1	0	0	0	1	0
Total	249	65	542	449	704	448

Differentially expressed PRGs were divided into 22 functional categories, and definitions can be found at http://prgdb.crg.eu/wiki/.

## Discussion

In the last two decades, molecular mechanisms involved in trichome formation have been investigated for the purpose of analyzing the factors controlling cell fate and plant cell differentiation^6,15^. However, the molecular regulation basis of multicellular trichome formation in pepper remains elusive. Similar to trichomes in tomatoes, pepper trichomes are multicellular structures. GZZY-23 is a hairy pepper variety with a high density of long non-glandular trichomes on the leaves and stems, and a few short glandular trichomes (Fig. 1A,B). PI246331, on the other hand, has sporadic short trichomes on the stem, showing a hairless phenotype (Fig. 1B). Therefore, these two materials are ideal candidates, making it easier to study the formation and development of multicellular trichomes.

RNA-Seq has become one of the most widely used tools based on next-generation sequencing technology, and has been used to analyze transcriptional regulation during trichome formation in plants such as tomato, cucumber, and tobacco^33,57,58^. The proportion of full-length transcripts from Illumina RNA-Seq assembly is small, and inaccuracy in gene structure characterization resulting from misassembly is a common problem^44^. SMRT sequencing developed by PacBio can help achieve full-length sequencing without full-length post-sequencing assembly, and the error rate of PacBio reads can be overcome by correction with Illumina RNA-Seq^42^. Thus, the hybrid approach combining both short-read next-generation sequencing technology and long-read SMRT sequencing, as a high throughput and cost-effective approach of sequence determination, has considerably improved the quality, efficiency, and speed of gene discovery. In the present study, transcriptome sequencing and analysis were performed to reveal the molecular mechanisms of pepper trichome formation using a hybrid approach combining both Illumina- and SMRT-based RNA-Seq; 40,079 genes were obtained, which contained 35,336 previously annotated genes and 4,743 novel genes (Tables 2 and S5). Since there are many gaps in the Zunla-1 genome data, it cannot effectively predict all transcripts, especially long transcripts. However, long transcripts can be efficiently obtained using SMRT sequencing. Therefore, in this study, we found that SMRT sequencing produced more long transcripts, especially transcripts larger than 2 kb, compared with those produced by the reference genome (Table 1). A previous study showed that lncRNAs are involved in the regulation of trichome formation^59^. Therefore, it was necessary to screen lncRNAs and determine their possible roles in pepper trichome initiation. Here, 2,227 lncRNAs were identified based on PacBio sequencing data (Table S7). AS and APA are two ubiquitous post-transcriptional regulations that could generate transcriptome and subsequently proteomic diversity in eukaryotes^60^. PacBio sequencing has enabled the identification of the complexity of AS in plants at the genome-wide level and the identification of cleavage sites for polyadenylation, which is important for gene annotation^43,61^. Thus, using PacBio sequencing, we obtained 4,427 AS events in this study (Table S6). It was possible that these differential splicing-related genes are involved in the splicing regulation during trichome formation. PacBio sequencing was also used for obtaining long reference sequence and for profiling 10,327 APA sites from 5,947 genes in pepper (Table S8). The data presented a comprehensive view of APA at the genome-wide level.

Previous studies have showed that many genes essential for multicellular trichome formation were highly expressed in hairy materials, especially in trichomes, such as *Wo*, *SlCycB2*, *SlCycB3*, *NtCycB2*, *H*, and *CaH*^25,32,33^. Thus, according to the comparative analysis of the three groups, DEGs highly expressed in trichomes might participate in multicellular trichome formation in pepper. Here, 1,038 overlapping DEGs were identified in the three comparisons (Fig. 2C). Of which, 521 and 474 DEGs were most highly expressed in GZZY-23 and Trichome, respectively (Tables S9, S10, and S11). Interestingly, many homologs of known trichome regulators were also highly expressed in GZZY-23, especially in trichomes, such as *CaH* and *CaCycB2* (Fig. 6; Tables S9, S10, and S11).

The formation of multicellular trichomes may be controlled in a distinct pathway from unicellular trichomes. However, multicellular trichomes in Solanaceous species may share a common regulatory pathway, as they are mediated by HD-ZIP IV, SlCycB2, and C2H2^26^. In the present study, the homologs of *SlCycB2* and *H* were also found to be significantly differentially expressed in the three comparisons. *CaCycB2* (Capana10g002051), *CaCycB3* (Capana06g000649), and *CaH* (Capana00g000811) were upregulated in GZZY-23, especially in trichomes (Fig. 6; Tables S9, S10, and S11). Many genes belonging to the HD-ZIP transcription factor family were highly expressed in GZZY-23, especially in trichomes (e.g., *Capana01g001442* and *Capana03g001526*) (Table S15). Therefore, genes involved in pepper trichome formation may also function in the formation of trichomes in other Solanaceae members.

Several studies have reported that transcription factors are involved in trichome formation, including MYB, bHLH, HD-Zip, and C2H2^25,26,58^. In this study, transcription factor activity, sequence-specific DNA binding (GO:0003700), and sequence-specific DNA binding (GO:0043565) were obviously enriched (Fig. 3A). Moreover, 11 DEGs involved in trichome differentiation (GO: 0010026) and trichome morphogenesis (GO: 0010090) were identified in the three comparison groups (Table S14). Of these, *Chr06.1236*, *Capana00g002415*, and *Capana00g003240* belonged to the MYB family. *Capana07g001455* encoded the HD-ZIP transcription factor (Tables S9, S10 and S11). Here, 312 TFs belonging to the bHLH, bZIP, C2H2, MYB, and HD-Zip families were significantly upregulated or downregulated in GZZY-23 or Trichome (Table S15). The differentially expressed TFs highly expressed in trichomes were identified, and most of these TFs showed a higher expression in GZZY-23 than in PI246331, suggesting that they possibly participate in the regulation of multicellular trichome formation, such as *CaH* (Figs. 5 and 6). Interestingly, some TFs that were highly expressed in GZZY-23 were downregulated in trichomes, suggesting that these genes may regulate trichome initiation (Fig. 5; Table S15). Studies have shown that the formation and development of trichomes are regulated by hormonal signals such as the GA, CK, and IAA signals^15,26^. The initiation of trichome development can be induced in the glabrous GA-deficient mutant *gal-3* by applying exogenous GA^57^. The application of CK resulted in the formation of trichomes on the stem of the inflorescence^62^. The suppression of *SlIAA15* in tomato can reduce the formation of trichomes, and this demonstrated that auxin is also required for multicellular trichome initiation^63^. Many genes related to the signaling pathway of phytohormones were also changed in the present study. We identified 151 DEGs involved in hormone signaling (Fig. 7). Most of those involved in SA, ABA, and ETH were upregulated in trichomes (Fig. 7).

Plant trichomes frequently function as the first line of defense against biotic and abiotic stresses through physical and/or chemical resistance^26,64,65^. Overexpression of a weed (*Solanum americanum*) proteinase inhibitor *SaPIN2a* in tobacco increased glandular trichome density and branches, which enhanced resistance to *Helicoverpa armigera* and *Spodoptera litura*^65^. In addition, overexpression of *Wo* in tobacco not only induced the formation of trichomes, but also increased plant resistance to aphids, suggesting that leaf trichome density positively correlated with herbivore resistance^26^. GO enrichment analysis showed that response to stress (GO:0006950), defense response (GO:0006952) and chitin metabolic process (GO:0006030) were enriched by upregulated DEGs in the comparisons PI246331 vs. GZZY-23 and PI246331 vs. Trichome (Fig. 3A,B and Table S12). Moreover, in our KEGG pathway analysis, upregulated DEGs were predicted to function in MAPK signaling pathway-plant (ko04016) and plant-pathogen interaction (ko04626) in the three comparisons (Fig. 4 and Table S13), which was similar to the results of previous studies^26^. We thus speculate that the trichomes in GZZY-23 leaves and stems (Fig. 1A,B) may increase the resistance to herbivores. Previous studies demonstrated that hormones not only participate in trichome development, but also play an essential role in plant defense against herbivores^15,26,57,62,63,66^. Here, numerous genes related to ABA, ETH, JA, and SA signaling pathway were significantly upregulated in GZZY-23, especially in trichomes (Fig. 7), which may also contribute to herbivore resistance in pepper. Moreover, we found that the expression level of many genes participating in chloroplast formation and photosynthesis was repressed in GZZY-23, especially in Trichomes (Table S9, S10, S11). Therefore, it can be implied that the photosynthetic activity in GZZY-23 and Trichomes may be insufficient. Likewise, the declined photosynthetic activity in tobacco plants with more trichomes was observed due to quick cell proliferation in multicellular trichomes^26^.

## Conclusions

To our knowledge, this is the first study to perform comparative transcriptome analysis between two *Capsicum annuum* L. cultivars to identify biological processes and functional gene activities involved in trichome development. We screened 312 DEGs that may be involved in trichome formation, belonging to the bHLH, MYB, HD-ZIP, and zinc finger protein families. Moreover, trichome regulators *CaCycB2*, *CaCycB3*, and *CaH* were considerably upregulated in the cultivar GZZY-23, especially in trichomes. The DEG analysis of plant hormone signal transduction indicated that plant multicellular trichome development may be mainly dependent on the IAA and CTK signaling pathways, and plant stress may be dependent on the ETH, ABA, and SA signaling pathways. In addition, 1629 DEGs were predicted as PRGs. Most of these genes were upregulated in GZZY-23 or trichomes. An extensive characterization of pepper trichomes will not only help understand the underlying molecular mechanisms involved in multicellular trichome development but also pave the way for creating new pepper varieties with desired trichome growth and density.

## Materials and methods

### Plant materials and growth condition

Pepper varieties GZZY-23 and PI246331, with and without trichomes, were used in this study. GZZY-23, a native variety, was collected by Yanxu Yin in Guizhou Province, China in 2014. PI246331 was provided by United States Department of Agriculture (USDA) (https://npgsweb.ars-grin.gov/). Both of them were grown in an intelligent green house with temperature regimes of 24–28 °C day/20–25 °C night, photon flux density of 300–350 μmol m^−2^ s^−1^, and relative humidity of approximately 70–80%. In the four leaf stage, images of the samples were captured using HUAWEI P30 Pro, and the stems were collected for SEM. The growth tips of GZZY-23 and PI246331 with two young leaves and GZZY-23 trichomes were used for RNA-Seq. After freezing in liquid nitrogen, the trichomes were brushed from the growth tip of GZZY-23 and two young leaves. The GZZY-23 trichomes were prepared by mixing trichomes of 60 seedlings. Other samples were composed of samples from five plants. Three biological replicates were used for each treatment for sequencing. All samples were collected at the same time, ground into powder in liquid nitrogen, and stored at − 80 °C for further use.

### Scanning electron microscopy

The pepper stems were detached and immersed in formaldehyde acetic acid–ethanol (FAA) containing 50% (v/v) ethanol, 5% (v/v) acetic acid, and 3.7% (v/v) formaldehyde. After 24 h fixation, the leaves were dehydrated in a graded ethanol series, and then critical-point dried by a desiccator (HCP-2, Hitachi). The samples were then sputter coated with gold palladium using Hitachi E-1045 ion sputter and carbon coating unit, and observed under a JSM-6390/LV scanning electron microscope, following the method of Gao et al^33^.

### Library construction and sequencing

Total RNA was extracted from the trichomes of young primary leaves of PI246331, GZZY-23, and GZZY-23 using TRIzol (Invitrogen, California, USA) according to the manufacturer's instructions, and then treated with DNase I (Promega, Beijing, China) to eliminate genomic DNA contamination. We used 1.2% agarose gel electrophoresis and a Nanodrop 2000 spectrophotometer (Thermo Scientific, DE, USA) to check the integrity and concentration of all nine RNA samples. The RNA samples were sent to Frasergen Bioinformatics Co., Ltd. (Wuhan, China) to generate libraries for sequencing. The nine libraries were constructed as described previously^36^, with minor modifications. Briefly, the first strand cDNAs were synthesized from the total RNA with random hexamer primers. The second stranded cDNAs were synthesized using DNA polymerase I (Biolabs, New England) and RNase H (Invitrogen). After end repair/dA-tail and adaptor ligation, the suitable fragments of double-strand cDNA were isolated by Agencourt AMPure XP beads (Beckman Coulter, Inc.), and then enriched by PCR amplification. Thereafter, Agilent 2100 Bioanalyzer (Agilent Technologies, Inc., CA, USA) and Qubit 2.0 were used to measure the purity and quality of the libraries. The nine cDNA libraries prepared were then sequenced by Biomarker Technologies (Wuhan, China) using the Illumina HiSeq 2500 platform. At the same time, a single sample was obtained by mixing the samples of PI246331 and GZZY-23, and was used for PacBio library preparation according to the manufacturer's instructions. Finally, the library was sequenced using Iso-Seq with PacBio RS II systems (Pacific Biosciences, Menlo Park, CA, USA). Raw sequence data are deposited in the Short Read Archive (SRA) of National Center for Biotechnology (NCBI) and are available under accession number PRJNA658737.

### RNA-Seq data and differential expression analysis

The raw RNA-Seq data obtained after sequencing of cDNA libraries were quality checked with the FastQC package to eliminate low quality reads with only adaptor, unknown nucleotides of > 5%, or Q20 of < 20%. The high-quality clean reads screened from raw reads were mapped independently to the reference genome *Capsicum annuum* L. Zunla-1 (https://www.pnas.org/content/111/14/5135) with TopHat 2.0 software^67,68^. Fragments per kilobase of transcript per million fragments mapped read values were used to calculate the mapped clean reads of each gene based on Cufflinks software^69^. DEseq2 was used to identify the DEGs among PI246331, GZZY-23, and Trichome^56^. The gene abundance differences among PI246331, GZZY-23, and Trichome were calculated by the ratio of the FPKM values. The significance of gene expression level was characterized by the FDR values as standards. The genes with an absolute value of |log2(fold change)|≥ 1 and FDR significance score < 0.05 were recognized as significant DEGs, and were used for further analysis. In addition, SMRT Analysis v2.3.0 was applied to analyze the clean reads of SMRT-based RNA-Seq data, as in a previous study^70^.

### GO and KEGG enrichment analyses

To characterize the putative functions of the DEGs, classification and enrichment of DEGs were carried out using the online tool ArigGO (GO Analysis Toolkit and Database for Agricultural Community)^71^. Pathway enrichment analysis was performed using the KEGG orthology database (http://www.genome.jp/kegg) with KOBAS2.0^72–74^. The GO and KEGG pathway enrichment analyses were conducted using a hypergeometric test and the Benjamini–Hochberg FDR correction (FDR ≤ 0.05).

### Quantitative RT-PCR (qRT-PCR) analysis

The relative expression levels of DEGs collected independently were confirmed by qRT-PCR to validate the results of the DEGs analyses. Approximately 2 μg of total RNA was isolated from young leaves of PI246331, GZZY-23, and trichomes with TRIzol reagent, which was used to synthesize the cDNA using the cDNA synthesis kit (TransGen, Beijing, China) according to the manufacturer’s instructions. We performed quantitative real-time PCR (qRT-PCR) in 96-well plates on Thermo Fisher Scientific Biosystems QuantStudio 5 Real-Time PCR system (Applied Biosystem, MA, USA) using the SYBR Premix Ex Taq Kit (Takara, Dalian, China). Ubiquitin-conjugating protein CaUbi3 (Accession Number: AY486137.1) was used as the internal reference^75^. Three independent biological replicates were analyzed. The relative expression level of the selected genes was calculated using the 2^-∆∆CT^ method^76^. All primers for qRT-PCR were designed according to the transcript sequences using Primer Premier 5.0 and the primers used in this experiment are listed in Table S17.

### Identification of AS events and lncRNAs

AS events were identified using ASTALAVISTA program from Illumina and SMRT RNA-Seq data in this study. The Cuffdiff tool was used to identify these AS events using transcript models obtained from cufflinks^77^. The transcripts were excluded, which displayed a read number of less than 25% uniquely mapped reads compared with that of the surrounding exons, insufficient read coverage (read number < 30) at junction sites, and putative AS events that would lead to very short proteins in the alternate ORF^78^. Moreover, the SMRT RNA-Seq data were used to identify lncRNAs as described previously^79^. After discarding the transcripts without strand information and transcripts that overlapped with known genes, the transcripts of longer than 200 nucleotides with FPKM higher than 0.5 in multiple-exons or 2 in single-exon in at least one sample were screened. The coding potential of the remaining transcripts was predicted using the coding potential calculator (CPC) and coding-non-coding index (CNCI) program; the transcripts with CPC scores ≤ 0 or CNCI ≤ 0 were retained as candidate lncRNAs.

### Identification of TFs and PRGs

TFs, like MYB, bHLH, HD-ZIP, and zinc finger proteins were identified using PlantTFDB (http://planttfdb.gao-lab.org/), which included the sequences of 58 transcription factor families from 165 plant species^80^. The UniGene sequence was compared with the transcription factor database by Blastx alignment and the gene with the best E value < e^−5^ was selected as the annotation information for the UniGenes.

There were more than 112 resistance genes and 104,335 candidate resistance genes in the Plant Resistance Genes database (http://prgdb.crg.eu/wiki/) ^81^. The sequences of UniGenes were compared with those in the PRG database by Blastx alignment, and the best of these with an E value < 10^–5^ was screened as the annotation information of the UniGene.

## Supplementary Information

Supplementary Tables.

## Data Availability

The collection of seed complied with local and national guidelines and permissions of seed were obtained. The RNA-Seq data supporting the results of this article have been uploaded to the Sequence Read Archive of NCBI (National Center for Biotechnology Information). It could be accessed via the NCBI SRA database with accession numbers of PRJNA658737.
